# Preliminary and ongoing French multicenter prospective naturalistic study of adverse events of antipsychotic treatment in naive children and adolescents

**DOI:** 10.1186/1753-2000-8-18

**Published:** 2014-06-13

**Authors:** Marie-Line Menard, Susanne Thümmler, Philippe Auby, Florence Askenazy

**Affiliations:** 1 University Department of Child and Adolescent Psychiatry, Nice Children's Hospitals CHU-Lenval, 57 avenue de la Californie, 06200 Nice, France; 2 Paediatrics and CDC, Lundbeck SAS, 92445 Issy-les-Moulineaux, France

**Keywords:** Adverse drug events, Antipsychotics, Therapeutic drug monitoring, Drug-naïve population, Child psychiatry, Pediatrics

## Abstract

**Background:**

The prescription of antipsychotics (AP), and especially second generation AP, is increasing worldwide in the pediatric population. Most prescriptions are off-label and despite the identification of frequent and potentially severe adverse events (AE), there are only a few guidelines for the safety management. France is one of the countries with no official safety guidelines.

**Methods:**

Psychotropic drug-naive adolescents (13–18 years), hospitalized for an acute psychotic episode and treated with a second-generation antipsychotic were consecutively included in a prospective cohort study. Patients were assessed for their AE at baseline, 2, 6 and 12 weeks after the introduction of drug.

**Results:**

The majority of patients was treated with risperidone (n = 13), 2 with aripiprazole. The principal findings are: (1) A high incidence of neuromuscular AE: 8/15 muscle weakness, 8/15 extrapyramidal syndrome, 6/15 akathisia, 3/15 oro-facial acute dystonia; (2) Severe catatonia symptoms in 2 patients despite a low to moderate treatment dose, requiring transfer in intensive care unit for one; (3) Weight gain and significant increase of the BMI for all 13 patients who had a 12 weeks follow-up.

**Conclusion:**

All adolescents experienced AE, with significant weight gain being observed in all patients who completed the 12-week follow-up. The fact that our patient population was first episode drug naïve may partially explain this observation. Despite the limitation due to the small sample size of this prospective short-term study, such findings are important to report and warrant further research.

**Clinical and research implication:**

Because of the lack of naturalistic follow up studies of antipsychotic treatments in AP-naive children and adolescents and the absence of safety guidelines for the pediatric population in France, we decided to continue our research at a national level. We therefore started a prospective, naturalistic and multicenter study funded by the French National Agency for Medicines and Health Products Safety (ANSM). Study purpose is to evaluate the incidence of adverse events related to antipsychotic drugs in AP-naive children and adolescents. In addition, we aim to provide further evidence for the necessity of national safety guidelines for AP prescription in the pediatric population.

## Background

The prescription of psychotropics has increased in the pediatric population all over the world since about 15 years. This increase is widely varying depending on the country and molecules, ranging from 1.5- to 5-times in many European countries and the United States [1,2]. In France the frequency of annual psychotropic prescriptions in children and adolescents is about 2.2% [2]. The increase in antipsychotic (AP) use is explained by a dramatic increase in atypical or second-generation AP (SGA) use, while typical or first-generation AP (FGA) prescriptions decreased [3]. SGA have a comparable efficiency to FGA with better neuromuscular safety [4]. Many AP medications in the pediatric population are prescribed off-label, especially in very young children [5,6]. Prescriptions are therefore frequently unsupported by rigorous scientific evidence. SGA drugs are actually widely used to treat many psychiatric disorders such as schizophrenia, bipolar, autistic spectrum, attention deficit hyperactivity or behavior disorders in the pediatric population [7-9]. However, the pediatric literature data show several adverse events (AE) in children and adolescents treated with AP [10-14]: muscular (muscle weakness, extrapyramidal syndrome, akathisia, dystonia, dyskinesia, catatonia), metabolic (weight gain, obesity, dyslipidemia, hyperglycemia, diabetes, insulin resistance, hypertriglyceridemia, hypercholesterolemia) and endocrine AE (hyperprolactinemia, vitamin D deficiency). Children treated with SGA are more likely than adults to experience AE. Woods et al. [15] studied more than 4 million prescriptions of olanzapine. They observed that the relative risk of sedation, weight gain, dystonia and tardive dyskinesia is 2 to 5 times greater in children or adolescents than in adults. Additionally, only few prospective studies examine antipsychotic adverse events in naive pediatric populations [16-18]. Several European and American authors are worried about long-term effects of antipsychotic medication on children’s health and recommend more safety guidelines and management of adverse events [19-26]. In fact, there are currently few guidelines for the management of adverse events in youth treated by antipsychotics [27-29]. On the European level, the lack of official guidelines for standardized follow-ups in the pediatric population is surprising given the probably higher relative risk of AE in this population. Furthermore, the incidence of AE in a drug-naive population is less well documented, as the typical study populations consist of pediatric patients who have already been exposed to an antipsychotic drug [30,31]. In France, since 2007 only two second-generation antipsychotics have been granted market authorization: risperidone for severe behavioral disorders from 5 years of age; and aripiprazole for schizophrenia from the age of 15 years and recently for maniac episodes associated with bipolar I disorder from the age of 13. The frequency of antipsychotic off-label prescriptions is as high as 69% in French university pediatric hospitals [32]. In March 2010, the French National Agency for Medicines and Health Products Safety (Agence nationale de sécurité du médicament et des produits de santé, ANSM) recommended cardiometabolic monitoring for all adult subjects treated with psychotropic drugs. There is no official recommendation for the safety monitoring in the pediatric population. This is paradoxical, since this population is at high risk of adverse events. In addition, adverse events during AP use in children are often poorly and insufficiently monitored in France in general practice.

## Clinical studies

### Preliminary study

In our own clinical experience, based on a university inpatient unit specifically aimed to treat young adolescents with a first psychotic episode, we have observed that adverse events (AE) such as weight gain, acute dystonia and catatonia were very common in these patients treated for the first time with SGA. We therefore undertook a naturalistic prospective cohort study in the University Children and Adolescent Psychiatric Department of Nice, France, in order to evaluate the incidence of AE related to SGA and we present the main results observed in our clinical population. This psychiatric department is the only inpatient adolescent psychiatric facility serving the one million and a half inhabitants of the French department of the Alpes-Maritimes. Inpatient psychotropic naive adolescents presenting a first acute psychotic episode and treated with a SGA were consecutively included in the study over a 12-month period from July 2009 to July 2010. Each patient was followed for 12 weeks.

**Figure 1:**
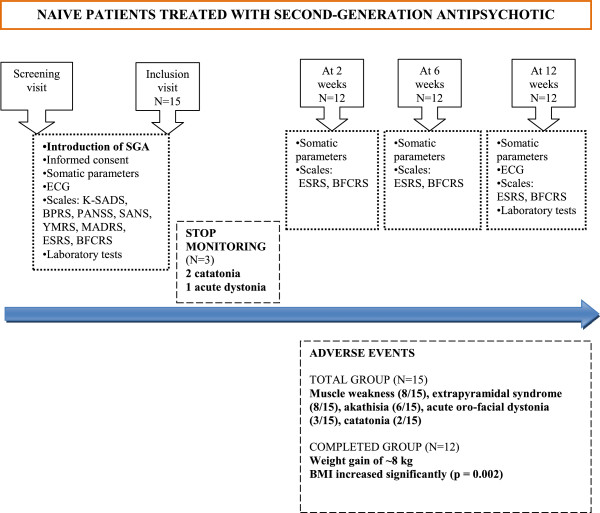
**Flow chart preliminary study.** Flow chart of the preliminary study (Assessment of adverse events in a naive pediatric population presenting psychosis and treated with a second-generation antipsychotic). A prospective study with a 12-weeks follow-up. BFCRS: Bush Francis Catatonia Rating Scale; BPRS: Brief Psychiatric Rating Scale; ECG: Electrocardiogram; ESRS: Extrapyramidal Symptom Rating Scale; K-SADS: Schedule for Affective Disorders and Schizophrenia for School Age Children; MADRS: Montgomery and Asberg Depression Rating Scale; PANSS: Positive and Negative Syndrome Scale; SANS: Scale for the Assessment of Negative Symptoms; SGA: Second-Generation Antipsychotic; YMRS: Young Mania Rating Scale.

The study was approved by Lenval Children's Hospital Scientific Committee. The following inclusion criteria were used: (1) male or female inpatient; (2) aged between 13 and 18 years; (3) requiring a SGA treatment; and (4) never before treated with any psychotropic medication. The follow-up included 4 visits: at baseline (W0), in the 48 hours before the introduction of the SGA, at 2 (W2), at 6 (W6), and at 12 weeks (W12) after the introduction of the SGA (Figure 1). The same investigator who was experienced in the use of the standardized rating scales assessed all adolescents. The clinical diagnosis was confirmed by Kiddie-SADS (2002 version), and several psychometric assessments were performed: Brief Psychiatry Rating Scale (BPRS), Positive and Negative Syndrome Scale (PANSS), Scale for the Assessment of Negative Symptoms (SANS), Young Mania Rating Scale (YMRS), Montgomery and Asberg Depression Rating Scale (MADRS) [33-37]. Neuromuscular AE were documented with the help of the Extrapyramidal Symptom Rating Scale (ESRS) and the Bush Francis Catatonia Rating Scale (BFCRS) [38,39]. All laboratory assessments were performed by the same university department laboratory (complete blood count, liver enzymes, creatine phosphokinase, fasting glycemia, cholesterol (HDL, LDL, total), triglycerides, and prolactin) at baseline and at W12. Fifteen adolescents, 8 males and 7 females, were included. Their average age (±SD) was 14.9 ± 1.38 years. Thirteen were treated with risperidone, and two with aripiprazole. The average oral dose was 2.5 ± 1.6 mg/d for risperidone and 7.5 ± 2.4 mg/d for aripiprazole throughout the follow-up. At baseline, DSM-IV-R diagnoses were: acute psychotic episode (15/15), schizophrenia (8/15), mood disorder (3/15), severe anxiety disorder (2/15) and borderline personality disorder (2/15).

We observed a high incidence of neuromuscular AE during the follow-up, leading to the discontinuation of AP treatment (risperidone) in 3 patients (catatonia 2/3, acute oro-facial dystonia 1/3). Muscle weakness was reported in 8/15 subjects, extrapyramidal syndrome in 8/15, akathisia in 6/15 and acute oro-facial dystonia in 3/15.

Severe catatonia symptoms were observed in 2 patients with paranoid schizophrenia at the 4^th^ and 7^th^ day of treatment while receiving a low to moderate dose of risperidone (1.5 and 2 mg respectively). Both patients presented stupor, mutism, staring, catalepsy and rigidity. In addition, verbigeration and withdrawal with refusal to eat and drink was a clinical feature in the first patient, and echolalia and impulsivity in the second one. Patients did not have fever or dysfunctions of the autonomic nervous system. They presented high scores on the Bush Francis Catatonia Rating Scale (17 and 12 respectively). SGA was immediately stopped and patients received clonazepam at dose 0.05 mg/kg/d. The first patient had to be transferred to the intensive care unit for hypoglycemia and dehydration in order to receive intravenous treatment.

One patient presented acute oro-facial dystonia. AP treatment was stopped and the patient received anticholinergic medication (one dose of intramuscular tropatepine 10 mg).

Moderate akathisia was present in 6 patients, sometimes starting as early as 2 weeks of SGA treatment. Severity was evaluated using the Extrapyramidal Symptom Rating Scale (ESRS).

For all patients - with the exception for one patient who developed catatonia - creatine phosphokinase was normal during follow up despite of a significant increase (p = 0.03, Wilcoxon test). No correlation was detected between this increase and neuromuscular AE (muscle weakness, extrapyramidal syndrome, akathisia).

The 12 patients who completed the 12 weeks follow-up presented a significant weight gain with 51.1 ± 8.5 kg at W0 and 59.0 ± 11.2 kg at W12 (p = 0.002, Wilcoxon test). The BMI increased significantly from 19.0 ± 2.5 at W0 to 21.7 ± 3.5 at W12 (p = 0.002). The observed weight gain of about 8 kg during 12 weeks is higher than in two other pediatric studies of comparable duration (3,9 et 5,3 kg for risperidone [40,30]; 4,4 kg for aripiprazole [30]; this might be explained by the small subject number or dose differences in our study.

No abnormalities were observed for the other monitored parameters, including lipid values.

In summary, we observed in our study many and serious adverse events despite the small sample size of 15 subjects. The particularity of this study is that all patients presented a first psychotic episode and had never been exposed to antipsychotic and/or other psychotropic medications before, which might partially explain this observation. For all these reasons and because of the lack of naturalistic follow-up studies of antipsychotic treatments in naive children and adolescents in France, we wanted to continue studying at the national level.

### A multicenter prospective naturalistic study in antipsychotic naive children (ETAPE Study)

**Figure 2:**
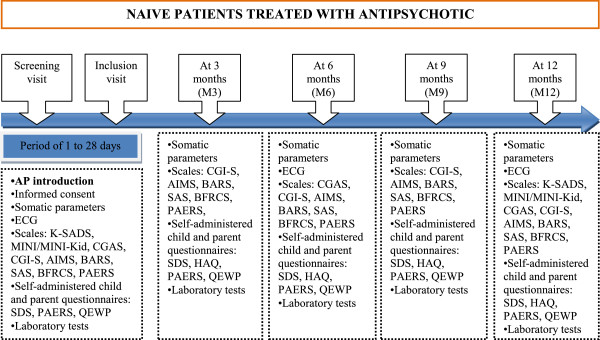
**Flow chart ETAPE study.** ETAPE Study (Assessment of adverse events in a naive pediatric population treated with an antipsychotic). A prospective multicenter naturalistic study with a 12-months follow-up which started in France in April 2013. AIMS: Abnormal Involuntary Movement Scale; AP: Antipsychotic; BARS: Barnes Akathisia Rating Scale; BFCRS: Bush Francis Catatonia Rating Scale; CGAS: Clinical Global Assessment Scale; CGI-S: Clinical Global Impressions Scales; ECG: Electrocardiogram; HAQ: Helping Alliance Questionnaire; K-SADS: Schedule for Affective Disorders and Schizophrenia for School Age Children; MINI: Mini International Neuropsychiatric Interview; PAERS: Pediatric Adverse Event Rating Scale; QEWP: Questionnaire of Eating and Weight Patterns; SAS: Simpson and Angus Scale; SDS: Sheehan Disability Scale.

In order to continue our safety monitoring project we have started a prospective, naturalistic and multicenter study to evaluate the incidence of adverse events related to the use of antipsychotic drugs in AP naive children and adolescents in France (**ETAPE**, **E**tude de la **T**olérance des **A**nti**P**sychotiques chez l’**E**nfant) [41,42]. This study is funded by the French National Agency for Medicines and Health Products Safety (ANSM, Agence nationale de sécurité du médicament et des produits de santé) and is registered on *ClinicalTrials.gov* (NCT02007928) [43]. The originality of this study lies in the inclusion of a AP naive pediatric population aged 6 to 18 years in 11 French pediatric psychiatry departments. Patients are included since April 2013 and the inclusion period will be for about 2 years with a follow up of each subject of 12 months. 340 patients should be enrolled in the study. The inclusion criteria are: (1) male or female, (2) aged from 6 to 18 years, (3) treated by antipsychotic drug, (4) never having received antipsychotic treatment before or having received AP for less than 3 months which has been discontinued 6 months prior to the study. Assessments are performed at the beginning of antipsychotic treatment and at 3, 6, 9 and 12 months after the introduction of antipsychotic drug (Figure 2). Diagnosis is made by the child psychiatrist and confirmed by the Kiddie - SADS scale (Schedule for Affective Disorders and Schizophrenia for School Age Children), M.I.N.I. (The Mini International Neuropsychiatric Interview) or M.I.N.I.-Kid (depending on the age of patient) based on DSM-IV-R criteria. An exhaustive investigation of AE is made using the Pediatric Adverse Event Rating Scale which has been designed for the systematic identification of AE in the pediatric population treated with psychotropics in clinical studies by the Child and Adolescent Psychiatry Trials Network [44] and translated in French by two different experts. Neuromuscular safety is assessed by the following clinical scales: Abnormal Involuntary Movement Scale (AIMS) [45] for dyskinesia, Barnes Akathisia Rating Scale (BARS) [46] for akathisia, Simpson and Angus Scale (SAS) [47] for extrapyramidal side effects, Bush Francis Catatonia Rating Scale (BFCRS) for catatonia. Several somatic parameters are monitored (weight, height, BMI, waist circumference, blood pressure, temperature, Tanner pubertal stage). On each visit, blood samples are analyzed for complete blood count, liver enzymes, creatine phosphokinase, fasting glycemia, cholesterol (HDL, LDL, total) and triglycerides. The study also includes a follow-up of thyroid function, prolactin, insulin, HOMA, HbA1C, vitamin D and C-reactive protein. Four self-administered questionnaires are proposed to child and parent: (1) Sheehan Disability Scale (SDS) for the evaluation of functional impairment in school, social and family life [48], (2) Helping Alliance Questionnaire (HAQ) to assess the relation between the patient, his family and the therapist [49], (3) Pediatric Adverse Event Rating Scale (PAERS) for the evaluation of side effects, (4) Questionnaire of Eating and Weight Patterns (QEWP) to specify eating disorders like bulimia or binge eating [50]. An electrocardiogram with the measurement of the QT interval is performed at baseline, 6 and 12 months.

## Conclusion

In the pediatric population, the use of antipsychotic drugs has been continously increasing over the last decade. In France, prescriptions are frequently off-label and starting more and more in young children. Adverse events are often not optimally monitored and without systematic follow-up. In addition, the lack of studies in children also leads to a safety concern.

The data of our preliminary study underline a high prevalence of neuromuscular AE in fifteen psychotropic drug naive inpatient adolescents treated with SGA for a first psychotic episode. We therefore started a multicenter prospective and naturalistic study to evaluate AP adverse events in naive children and adolescents in France. The results of ETAPE’s study will have a major impact in terms of public mental health services in our country and must help to develop official recommendations for antipsychotic treatment and its safety monitoring in the pediatric population.

### Implications and contribution

Beyond the available literature, more research is urgently needed about adverse events of antipsychotics in the pediatric population in order to develop official recommendations. However, enough is known to emphasize the necessity of thorough and systematic monitoring of AE in this vulnerable population. Furthermore, in the light of the growing trend to prescribe these drugs to children and adolescents, practitioners need to be aware of the necessity of optimal safety management.

## Informed consent

The study was approved by Lenval Children's Hospital Scientific Committee. Written informed consent was obtained from the patients and their parents.

## Competing interests

The authors have no conflicts of interest relevant to this article to disclose. Philippe Auby is a Lundbeck employee and the views presented in this article are those of the author alone, and should not be understood or quoted as being made on behalf of H. Lundbeck A/S or any of Lundbeck’s affiliate.

## Authors’ contributions

MLM is the principal author of the study, and guarantor for the results. FA and MLM contributed to the conceptualization of the study and data analysis. All authors contributed to the writing of the manuscript. All authors read and approved the final manuscript.
